# Forming Cardi-OH: A Statewide Collaborative to Improve Cardiovascular Health in Ohio

**DOI:** 10.7759/cureus.28381

**Published:** 2022-08-25

**Authors:** Shari D Bolen, Elizabeth A Beverly, Shireen Khoury, Saundra Regan, Jackson T Wright, Siran Koroukian, Randell Wexler, Goutham Rao, Daniel Hargraves, Dean Bricker, Glen D Solomon, Michael Holliday, Stacey Gardner-Buckshaw, Lance Dworkin, Adam T Perzynski, Elizabeth Littman, Ann Nevar, Shannon M Swiatkowski, Mary Applegate, Michael W Konstan

**Affiliations:** 1 Medicine, Case Western Reserve University at The MetroHealth System, Cleveland, USA; 2 Population and Quantitative Health Sciences, Case Western Reserve University School of Medicine, Cleveland, USA; 3 Cardiovascular Disease Programs, Better Health Partnership, Cleveland, USA; 4 Family Medicine, Ohio University Heritage College of Osteopathic Medicine, Athens, USA; 5 Family and Community Medicine, University of Cincinnati College of Medicine, Cincinnati, USA; 6 Medicine, Case Western Reserve University School of Medicine, Cleveland, USA; 7 Medicine, University Hospitals Cleveland Medical Center, Cleveland, USA; 8 Family Medicine, The Ohio State University Wexner Medical Center, Columbus, USA; 9 Center for Community Health Integration, Case Western Reserve University School of Medicine, Cleveland, USA; 10 Department of Family and Community Medicine, University of Cincinnati, Cincinnati, USA; 11 Medicine, Wright State University Boonshoft School of Medicine, Dayton, USA; 12 Family and Community Medicine, Northeast Ohio Medical University, Rootstown, USA; 13 Medicine, The University of Toledo Medical Center, Toledo, USA; 14 Center for Health Care Research and Policy, Case Western Reserve University at The MetroHealth System, Cleveland, USA; 15 Medicine, Case Western Reserve University, Cleveland, USA; 16 Internal Medicine-Pediatrics, Ohio Department of Medicaid, Columbus, USA

**Keywords:** social determinants of health (sdoh), cardiovascular disease (cvd), hypertension, primary care, quality

## Abstract

Background

Cardiovascular risk factor control is challenging, especially in disadvantaged populations. However, few statewide efforts exist to tackle this challenge. Therefore, our objective is to describe the formation of a unique statewide cardiovascular health collaborative so others may learn from this approach.

Methodology

With funding from the Ohio Department of Medicaid’s Ohio Medicaid Technical Assistance and Policy Program, we used a collective impact model to link the seven medical schools in Ohio, primary care clinics across the state, the Ohio Department of Medicaid, and Ohio’s Medicaid Managed Care Plans in a statewide health improvement collaborative for expanding primary care capacity to improve cardiovascular health in Ohio.

Results

Initial dissemination activities for primary care teams included a virtual case-based learning series focused on hypertension and social determinants of health, website resources, a monthly newsletter with clinical tips, webinars, and in-person conferences. The collaborative is aligned with a separately funded hypertension quality improvement project for paired implementation.

Conclusions

The collective impact model is a useful framework for developing a statewide collaborative focused on the dissemination and implementation of evidence-based best practices for cardiovascular health improvement and disparity reduction. Statewide collaboratives bringing payers, clinicians, and academic partners together have the potential to substantially impact cardiovascular health.

## Introduction

Cardiovascular disease (CVD) is the leading cause of death in the United States, with nearly half of Americans having major CVD risk factors (e.g., 45% hypertension, 10% diabetes, 14% smoking) [1-5]. In particular, blood pressure (BP) control is a major modifiable driver of improved cardiovascular outcomes and mortality [6-8]. However, only 45% of hypertensive patients in national samples achieve good BP control, even when defined as a BP less than 140/90 mmHg [3]. In addition, BP control among disadvantaged populations has been particularly difficult to achieve, as demonstrated by the persistent disparities in BP control by race/ethnicity and income [1,9,10].

Given the significant CVD morbidity and mortality and limited success in controlling specific CVD risk factors, we need mechanisms to accelerate the translation of cardiovascular evidence-based best practices into patient care to improve cardiovascular health outcomes [11]. Regional health improvement collaboratives have been a major way that geographic areas across the United States have brought together stakeholders to accelerate improvements and reduce disparities in health outcomes, including BP control [12-14]. These collaboratives are often guided by the “triple aim” of health system improvement: improving the health of populations, improving the experience of care, and reducing the per-capita cost of care [15,16]. While health collaboratives vary in clinical scope of interest and organizational structure, they share certain common characteristics, including operation on a non-profit basis, a core membership of credible stewards to assemble stakeholders within a focal geographic area, and the use of data and community engagement to understand, plan, and facilitate improvement in healthcare [13,16].

**Figure 1:**
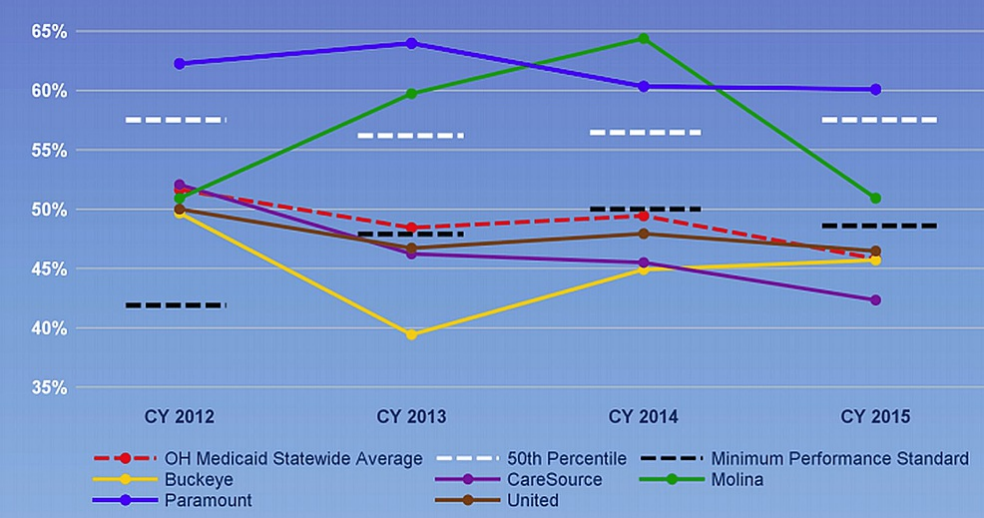
Ohio Medicaid controlling high blood pressure HEDIS measure overall and by Medicaid MCPs (2012-2015). CY: calendar year; HEDIS: Healthcare Effectiveness Data and Information Set; MCPs: managed care plans

In 2017, based on low rates of BP control from the Medicaid managed care plans (MCPs) Healthcare Effectiveness Data and Information Set measures [17] (see Figure 1 in Materials & Methods), the Ohio Department of Medicaid (ODM) decided to fund a statewide cardiovascular health improvement collaborative to focus on expanding primary care capacity to improve overall cardiovascular health and reduce disparities in cardiovascular health in the Medicaid population, with an initial focus on hypertension and addressing social determinants of health (SDOH) [18]. Unique to this collaborative was: (1) its statewide approach; (2) uniting the seven Ohio schools of medicine to serve as a backbone for the collaborative; (3) partnerships with networks of primary care providers for uptake of best practices into care; and (4) partnering with the Medicaid MCPs and ODM to leverage potential policy change. This collaborative approach presents a unique opportunity to accelerate the translation of best practices into care.

Although many regional health improvement collaboratives exist [13], only a limited number of statewide health collaboratives have been developed [19-23]. Of the statewide collaboratives, few have focused on both cardiovascular health improvements and reducing disparities in cardiovascular health [23]. To our knowledge, none of these cardiovascular-focused statewide collaboratives have included primary care clinics, payers, and academic medical schools as three key backbone organizations to drive subsequent cardiovascular health improvements. Therefore, we describe the formation of a unique statewide cardiovascular health collaborative and its initial planned activities so others may learn from our successes and challenges as they develop partnerships to accelerate the translation of evidence-based best practices into care.

## Materials and methods

Figure 1 presents the overall Ohio Medicaid controlling high blood pressure HEDIS measure overall and according to Medicaid MCPs from 2012 to 2015.

Description of collaborative formation

The Rationale for Initial Formation

Based on the goal of accelerating improvements in cardiovascular health and reducing disparities in cardiovascular health across the state, the ODM wanted to test a model where they funded the academic medical schools as a backbone organization for the development and dissemination of evidence-based best practices in partnership with primary care clinics and the Medicaid MCPs. Academic medical schools were chosen as the backbone for several reasons by the ODM: (1) academic primary care providers could help establish the evidence-based strategies to be used and promoted for cardiovascular health improvement; (2) academic medical schools are often seen as interprofessional conveners; and (3) many academic medical schools also have large affiliated primary care networks. The latter could potentially be used as test and reference sites to evaluate approaches to disseminating best practices. While professional organizations have large networks across the state, they are often focused on policy and advocacy work for their particular group and less able to serve as a neutral convener.

The collaborative was initially funded to focus on providing education to Medicaid primary care providers and their teams to better manage hypertension and address the SDOH. In conjunction with the collaborative, a separate Medicaid funding stream was established to support a hypertension quality improvement project (QIP) that would recruit and link primary care clinics with the academic medical centers, ODM, and the Medicaid MCPs. Ohio Medicaid MCPs are required to do performance improvement projects in two-year cycles, and they receive financial incentives when hitting specific quality metrics, one of which is BP control (rates of BP control to <140/90 mmHg) for their enrollees. Lessons learned from the hypertension QIP could then be disseminated more broadly across the statewide collaborative. If successful, this model might be used for other chronic conditions such as diabetes. Therefore, a request for applications (RFA) was developed by the ODM and sent to all seven schools of medicine to determine which school of medicine would lead this collaborative effort.

The Collaborative is Based on the Collective Impact Model

**Figure 2:**
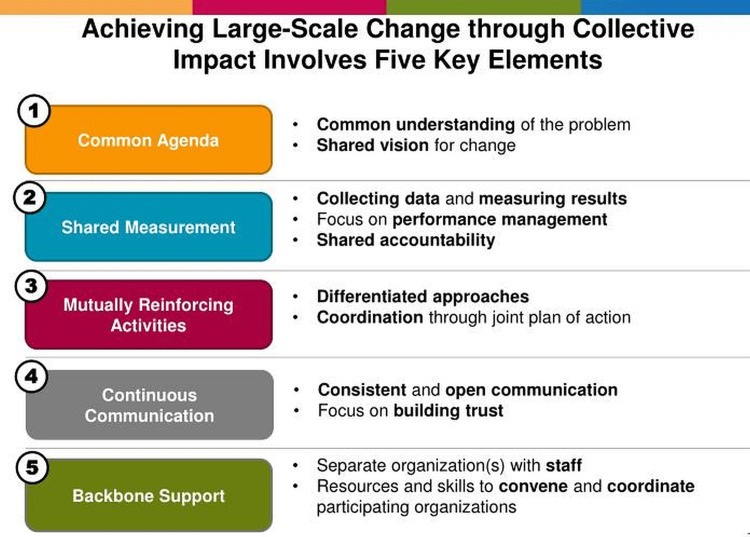
Five elements of the collective impact model. Adapted with permission from the authors [25].

While mainly used to drive social change, the collective impact model is a helpful framework when a large problem exists requiring multiple stakeholders to begin to make an impact such as improving CVD and reducing disparities in CVD across Ohio [24]. A collective impact model includes five key elements: (1) a common agenda, (2) shared measurement, (3) mutually reinforcing activities, (4) continuous communication, and (5) backbone support (Figure 2) [25]. The model typically has four phases that often transpire over three to five years: (1) generate ideas and dialogue, (2) initiate action, (3) organize for impact, and (4) sustain action and impact. As we engaged our members and developed our scope and activities, we conducted an initial strategic planning effort. For Phase 1 (generate ideas and dialogue), stakeholders met to develop a common understanding of the problem and shared vision for change. Our initial virtual meetings followed by an in-person kick-off helped establish our initial shared vision, mission, and high-level activities. For Phase 2 (initiate action), we developed a common agenda to share evidence-based best practices for hypertension management and SDOH, identify cross-sector champions, and begin outreach to primary care practices and medical school partners. For Phase 3 (organize for impact), we met virtually with stakeholders to finalize mutually reinforcing activities and shared success metrics. We developed an initial Charter to document our governance, organizational structure, vision/mission, shared metrics, and mutually reinforcing activities which was circulated to our partners for feedback and then finalized. For Phase 4 (sustain action and impact), we have begun implementing our dissemination activities (a case-based virtual learning series, webinars, in-person conferences, website resources, and a monthly newsletter), and are beginning to conduct sustainability planning. Continuous communication via our Cardi-OH monthly newsletter, webinars, and meetings are designed to build trust and provide consistent communication with our partners and members. The collective impact model assists collaboratives in avoiding coordination and cooperation challenges by continuing to meet with stakeholders to understand how their organizational priorities align with the collaborative.

## Results

Phase 1: engaging Ohio’s schools of medicine

Case Western Reserve University (CWRU) School of Medicine was selected as the lead institution based partly on preliminary data from a regional health improvement collaborative called Better Health Partnership, with over 400 primary care providers in the Northeast Ohio region which improved BP control and reduced disparities in BP control, including reducing disparities between Medicaid and Medicare patients and disparities in minority populations (compared to white populations) [14]. CWRU reached out to the other academic schools of medicine to assess their interest in participating in this statewide collaborative. In the initial application, CWRU included three of the six other schools of medicine as partners due to time, budget, and competitive constraints. On our initial phone calls, we outlined the draft high-level activities from the RFA provided by the ODM. Because the RFA was high-level, we had the capacity to work with our partners to develop activities to match the interests of our partners. Moreover, BP control was an important metric to many of our schools of medicine and their networks of primary care clinics.

Once funded as the lead organization, CWRU requested additional budget from ODM to support the inclusion of the other schools of medicine in the state. Five medical schools were funded in Year 1, and the remaining two schools joined the collaborative in Year 2 with additional funding. We allowed all of the medical schools to tailor their contributions to the collaborative based on their time, interest, and different areas of expertise to better engage them as partners.

Phases 1 and 2: developing the Cardi-OH Charter

During its initial planning phase, a Charter with a draft vision/mission, scope of work/activities, and organizational structure was drafted with the four initial partner schools of medicine. In initial meetings, the Executive Principal Investigator (PI) Team (leads from each of the medical schools) decided on several key activities to expand primary care team capacity to manage cardiovascular health by sharing best practices. We chose dissemination activities based on prior successful activities by our regional partners who had leadership roles in regional health improvement collaboratives and/or led successful dissemination projects in their medical schools. These key dissemination activities included: (1) the development of a Project ECHO series, a virtual case-based learning series to expand primary care capacity to manage cardiovascular health [26,27]; (2) an annual statewide in-person conference to network and share best practices around cardiovascular health and reducing disparities in cardiovascular health for Medicaid patients; and (3) webinars and website materials on cardiovascular health which could be shared with partners and members via a newsletter and e-mail. Due to budget restrictions, we were not able to fund quality improvement implementation activities within the collaborative; however, the collaborative was paired with a separate Medicaid-funded project to conduct a hypertension QIP within primary care practices across the state. Part of the collaborative’s charge was to align with those efforts, and several key collaborative leads served in lead roles for the hypertension QIP. Discussions on how best to align efforts occurred during the in-person kick-off and beyond. We decided during discussions to align efforts by: (1) assisting in creating educational content for the hypertension QIP when a need was identified; and (2) sharing best practices from the hypertension QIP with the larger collaborative primary care practice members to have a greater reach across the state.

**Figure 3:**
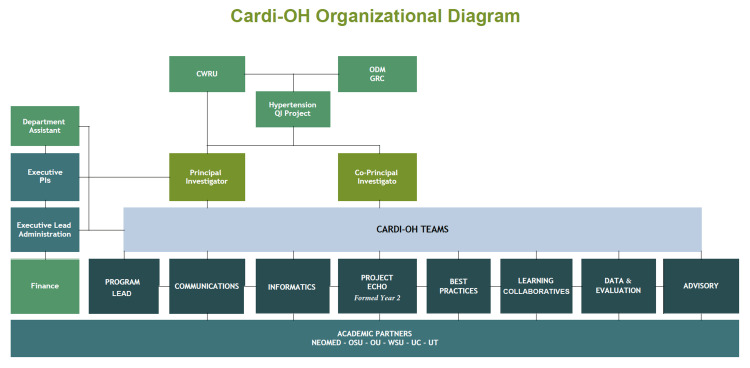
Cardi-OH organizational diagram. CWRU: Case Western Reserve University; GRC: Government Resource Center; NEOMED: Northeast Ohio Medical University; ODM: Ohio Department of Medicaid; OSU: Ohio State University; OU: Ohio University; QI: quality improvement; UC: University of Cincinnati; UT: University of Toledo; WSU: Wright State University

Due to the large number of people engaged in the collaborative initially (>80 people across the state), we needed to form teams to accomplish the scope of work. Therefore, the Executive Team worked with each medical school to create organizational charts (Figure 3). Seven teams were initially established, with team lead(s) for each: (1) Best Practices, to identify and/or develop high-priority educational topics based on best available evidence and national guidelines related to hypertension and SDOH for dissemination; (2) Data & Evaluation, to develop success metrics for the collaborative and evaluate different educational content/curricula being developed; (3) Learning Collaboratives, to establish a statewide conference in Year 1 to share best practices in cardiovascular health and SDOH; (4) Program Lead, to oversee day to day operations; (5) Communications, to create a communications plan including branding and logo, disseminating best practices activities, identifying strategic partners, working with media relations, planning and designing a website, establishing a Project ECHO Hub (a case-based learning series for primary care teams using a videoconferencing technology), and assisting in planning the statewide conference; (6) Informatics, to develop multiple online platforms for promoting and sharing best practices. This included developing and maintaining a website, creating and overseeing data management and shared storage systems, ensuring that the network and its partners had the information technology (IT) infrastructure required for the initiative; and (7) Advisory, to help provide high-level feedback on the collaborative goals and activities each year and advise or assist individual teams as needed. In Year 2, a Project ECHO Team was established to develop curricular content, recruit primary care providers, and run the Project ECHO series (two 12-week virtual case-based learning series around cardiovascular and SDOH topics). We asked each medical school partner to place team members within each of these teams to enhance statewide collaboration. All Cardi-OH team members are listed in Table 1.

**Table 1:** Cardi-OH collaborative team members and affiliations (2017-2018).

Affiliation	Team members
Case Western Reserve University School of Medicine	Michael Konstan, MD (PI); Shari Bolen, MD, MPH (Co-PI); Aleece Caron, PhD; Randall Cebul, MD; Richard Cornachione; Marco Costa, MD, MBA; Joseph DaPrano, MD; Pamela Davis, MD, PhD; Catherine Demko, PhD; Nicholas Dreher, MD; Douglas Einstadter, MD, MPH; Margaret Guncik; Rita Horwitz RN; David Kaelber, MD, PhD; Marc Kaplan; Shireen Khoury, MD, MPH; Siran Koroukian, PhD, MSN; Susan Krejci, MBA; Jessie Lewis, MPH; Elizabeth Littman; Lindsay Lodge; Chris Longenecker, MD; David Margolius, MD; Sarah McAleer, Med, RD; James Misak, MD; Shirley Moore, RN; Jeno Mozes; Suparna Navale, PhD; Ann Nevar, MPA; Devin O'Neill; Adam Perzynski, PhD; Ginny Petrie; Goutham Rao, MD; Tanya Robinson, RN, PhD; Martin Ryan, MD; Kelli Ryan, PhD; David Silvaggio; Mamta Singh, MD; Joseph Sudano, PhD; Catherine Sullivan, RD; Shannon Swiatkowski, MS; Kathryn Teng, MD, MBA; Patricia Thomas, MD; Daryl Thornton, MD; Teresa Uter; Phyllis Virgil MHA; Brooke Watts, MD, MS; Monica Webb-Hooper, PhD; James Werner, PhD; Khendi White-Solaru, MD; Jackson T. Wright Jr, MD, PhD; Ted Wymslo, MD; Amy Zack, MD
Northeast Ohio Medical University	John Boltri, MD (PI); Stacey Gardner-Buckshaw, PhD, MPA (Co-PI); Brian Bachelder, MD; Kris Baughman, PhD; Terri Christensen; Amy Lee, MD, MPH, MBA; George Litman, MD; Emily Murphy; Joseph Zarconi, MD
Ohio University	Elizabeth Beverly, PhD (PI); Sarah Adkins, PharmD; Darlene Berryman, PhD, RD, LD; Karie Cook, BSN, RN; Sebastian Diaz PhD, JD; Emily Guseman, PhD; Kenneth Johnson, DO; Rosellen Roche, MD; Tracy Shaub, DO; Melissa Standley; Jody Van Bibber; Stacy Wright, BSN, RN
The Ohio State University	Randell Wexler, MD, MPH (PI); Pamela Beavers; Anton Borja, DO; Cheryl Carmin, PhD; Aaron Clark, DO; Tamara Davis PhD, MSSW; Allard Dembe, ScD; Colleen Fitzgibbons, MPH; Mary Fristad, PhD; Kate Gawlik, DNP, RN; Iahn Gonsenhauser MD, MBA; William Hayes, PhD; Blessing Igboeli, MD; Katrina Johnson, MD; Deborah Larsen PhD; Jeffrey Lawrence, MD; Teresa Long, MD, MPH; John McConaghy, MD; Shalina Nair, MD, MBA; Adriane Peck; Kate Philips; Lisa Raiz, PhD; Mark Rastetter, MD; Kristen Rundell, MD; Stacey Saunders-Adams; Robin Shah, DO, MBA; Gbemiga Sofowora, MBChB, MSc; Chris Taylor, PhD; Alexa Valentino, PharmD; Mary Jo Welker, MD
University of Cincinnati	Michael Holliday, MD (PI); Sarah Brubaker; Bruce Gebhardt, MD; Daniel Hargraves, MSW; Joseph Kiesler, MD; Jacqueline Knapke, PhD; Christy O'Dea, MD; Harini Pallerla, MS; Saundra Regan, PhD; Anisa Shomo, MD; Barbara Tobias, MD; Mary Beth Vonder Meulen, RN; Christopher White, MD, JD, MHA
University of Toledo	Lance Dworkin, MD (PI); Basil Akpunonu, MD; Sarah Aldrich, PharmD; Marilee Clemons, PharmD; Jennifer Gilmore, RN; Nicholas Horen, MD; Sadik Khuder, PhD; Diane McCarthy, MPA; Marc Miller; Thomas Papadimos, MD, MPH; Zachary Phillips, MBA; Shipra Singh, MBBS, MPH, PhD; Ben Tobias, PA
Wright State University	Lawrence Lawhorne, MD (PI); Dean Bricker, MD; Jaycee Burgess; Roberto Colon, MD; Ronald Markert, PhD; Heather Maurer; Cynthia Sheppard Solomon, BSPharm, RPh; Glen Solomon, MD; Kerianne Springer, MD

In addition, PIs from each of the funded medical schools formed an Executive PI Team to steer the course of the collaborative and make critical decisions to meet the scope of work. Year 1 goals and deliverables with a timeline were drafted to accomplish the scope of work and engage and launch the collaborative’s activities.

An external consultant was hired to help with the development of the collaborative’s Charter and meet with key stakeholders at each school of medicine, ODM, and the Ohio Colleges of Medicine Government Resource Center which administers Medicaid-funded grants. The consultant synthesized comments from discussions outlining key activities collaborative members wanted to see happen with the collaborative’s launch. These personal, semi-structured interviews reinforced our initial activities and team structure, provided some direction to Team Leads, and enhanced the engagement of members across the collaborative. Subsequently, a Charter was drafted from this input. At an in-person, kick-off meeting with about 60 grant members from across the state, attendees met to refine and finalize the Charter, including the vision, mission, purpose, and goals (Table 2). This meeting allowed people to meet face to face who would be working together for the next few years. We used notes from the kick-off meeting to finalize the Charter for review and approval by Medicaid.

**Table 2:** Initial Cardi-OH vision, mission, and purpose.

Cardi-OH vision, mission, and purpose
Vision: For all Ohioans to reach their highest potential for cardiovascular health
Mission: To improve cardiovascular health outcomes and eliminate cardiovascular health disparities
Purpose: Expand the primary care team capacity to:
Prevent, diagnose, and manage cardiovascular disease in Ohio’s Medicaid population
Identify and address disparities in cardiovascular health care and outcomes affecting the Ohio Medicaid population

Phases 1 and 2: engaging stakeholders outside of the medical schools

Initially, we focused on bringing together the schools of medicine to build a strong foundation and backbone for the collaborative before moving forward. Therefore, our in-person kick-off comprised team members from the medical schools. We did have other key stakeholders on our advisory team or as part of our kick-off. For instance, ODM, the American Heart Association, primary care clinicians, and the chief medical officer for the statewide Ohio Association of Community Health Centers participated in providing their insight and feedback on our vision, mission, and activities. In subsequent years (Years 2 and 3), we began to expand our partners to engage with the Ohio Department of Health, Ohio Medicaid MCPs, professional organizations (e.g., the Ohio Academy of Family Practice), and employer groups. To engage with additional organizations or groups, we held one-on-one meetings initially to discuss potential shared goals and activities. We then invited them to participate in subsequent educational activities and in-person planning meetings.

Phases 1 and 2: engaging primary care teams in Cardi-OH

Because the PIs and teams at the medical schools incorporated primary care providers, we initially used the primary care networks within and associated with the medical schools. The medical schools were already partnered with three regional health improvement collaboratives comprising primary care practices, thus we had access to a large network of over 700 potential practices from which to disseminate materials across the state. These practices were our primary focus in the beginning. As we continue to move forward, we are looking to partner more closely with the Medicaid MCPs in Ohio and professional organizations to link websites and cross-promote via newsletters and e-mails.

Phase 3: establishing shared metrics, designing a tracking system, and monitoring progress

The goal of the Data Team was to establish a set of shared metrics to measure success (Table 3), design a tracking system, and monitor the progress of the Collaborative. All Team Leads participated in interviews to inform the set of metrics designed to track each team’s objectives and activities. These discussions determined the type of measure (e.g., needs assessment, satisfaction survey) and mechanism for collecting information (e.g., online anonymous survey, paper-and-pencil evaluation). All data were tracked in REDCap and managed by the Data Team in collaboration with the Informatics and Program Teams.

**Table 3:** Initial high-level success metrics, definitions, and tracking.

Success metrics	Definition	Tracking system
Medicaid providers reached	Number of Medicaid providers participating in Cardi-OH educational events	Registration at different educational activities
Cardi-OH content developed and used; satisfaction with materials	The number of website documents used by Cardi-OH members	Google analytics: number of hits to the website and each webpage, and geographic location of people accessing the website
	Other educational sessions and topics (e.g., webinars, in-person events)	Document the number of activities and topics; post-session satisfaction surveys
Uptake of best practices by Medicaid providers	Perception of uptake of best practices by primary care team members after educational events	Post-educational or dissemination event surveys

The first two success metrics (number of Medicaid providers reached, number of, and satisfaction with website education tools used) were more straightforward to measure than the third which involved the incorporation of best practices by Medicaid providers. At the end of Year 1, we did not yet have a mechanism to measure the uptake of best practices. In Year 2, we decided to measure whether attendees would implement something new based on the educational content they received in our post-educational event surveys.

Phases 2 and 3: engaging and communicating across teams

Because teams were meeting separately to accomplish the key activities, CWRU developed a monthly Team Leads meeting to promote cross-team engagement and alignment. In addition, Team Leads and Executive PIs exerted ongoing efforts for partners to participate on several meaningful levels to provide not only needed assistance but ownership of key components to achieving the objectives of the project overall. This was accomplished via a basic agenda with time allocated for introductions to new individual and institutional members and selection of working groups within their specialty per member discretion. A best practice identified within Team Best Practices includes requesting biosketches from members to optimize the performance of the group by recognizing and utilizing individuals’ expertise. All Team BP members were also strongly encouraged to participate in the review process for materials intended for dissemination. Lastly, when a member was not as engaged, we reached out to Executive PIs and the member to ask them about their interests to see if better alignment could occur to promote greater engagement.

## Discussion

We describe the formation of a unique statewide cardiovascular health collaborative (Cardi-OH) linking the seven academic medical schools, primary care clinics, and Medicaid payers, with the goal of improving cardiovascular health and eliminating disparities in the Medicaid population. Cardi-OH primarily focuses on the education of primary care teams via dissemination activities, but it is also closely aligned with a separately ODM-funded hypertension QIP to enhance the uptake of best practices into primary care. Having payers at the table has enhanced communication around insurance barriers that could be addressed to improve health outcomes, such as making it easier for patients to obtain home BP monitors at pharmacies and changing the 30-day prescription policy for Medicaid patients to 90-day prescriptions for hypertension management.

While many regional health improvement collaboratives exist which engage partners, disseminate best practices, and conduct quality improvement [13,14], few have developed statewide collaborative efforts. Ohio, Maryland, Georgia, Pennsylvania, and California have formed statewide collaboratives focused on key public health issues such as reduction of poor neonatal outcomes, reduction of early elective deliveries and caesarian section when not indicated, smoking cessation, drug overdoses, and reduction in alcohol use [19,21,22,28-31]. To our knowledge, these statewide initiatives have not focused on hypertension management or described the formation process for others to learn from their successes and challenges except for the Outpatient Quality Improvement Network (O’QUIN) [23]. The O’QUIN originating in South Carolina evolved from a hypertension initiative to a multi-state initiative aimed at the prevention of chronic disease. They described their unique formation as a practice-based improvement network through outpatient primary care clinics with audit and feedback approaches and multiple funding sources. Although highly successful and a wonderful example, their formation was quite different from what is described in this paper. Our formation was through the initial linking of the seven medical schools across the state and funded by ODM who brought the Medicaid MCPs to the table at initial formation for greater impact. While some lessons learned around trust and engagement are similar, we describe another important way a state can come together and collaborate to improve cardiovascular health, especially for disadvantaged populations.

Key lessons learned from our initial Cardi-OH formation included the following four principles: (1) build a culture of trust that values collaboration; (2) understand the motivation, interests, and strengths of partners; (3) optimize the collaborative’s needs with the strengths of partner institutions; and (4) communicate clear expectations and a timetable for collaborative milestones. Trust can be achieved when team members are given adequate time to cultivate meaningful relationships and develop mutual respect. Our strategic planning process with interviews followed by an in-person kick-off and subsequent charter with a shared vision, mission, and activities helped build the transparency and engagement important for trust building. A successful collaborative engages a wide range of partners and recognizes the valuable expertise provided by each partner. Including partners’ perspectives (e.g., motivation, interests, strengths) in the planning phase of the collaborative helped identify potential barriers, resources, and training needs for the future. The knowledge and experience of partners should be leveraged to translate, disseminate, and implement best practices into care; this process is ongoing with our annual needs assessment surveys. Collaboratives that understand the key attributes of their partners will place team members in roles that fully utilize their strengths to function optimally. To address this, we developed specific working groups/teams with specific team leads to meet routinely using the virtual platform. During the initial formation of Cardi-OH, the evaluation prioritized the process of implementation. This necessitated team leads to communicate clear expectations for process-oriented goals and celebrate the achievement of milestones as the collaborative progresses. After successful formation, the evaluation can focus on improved outcomes and overall effectiveness of the collaborative in the target population.

Our collaborative model comes with one clear limitation. Due to funding restrictions, we are unable to directly link our educational efforts to real-time clinical metrics from practices such as BP control. While we are aligned with the hypertension QIP which benefits from our collaborative and serves as a real-time opportunity to implement and evaluate the uptake of evidence-based best practices into care, we are unable to determine if the Cardi-OH educational events themselves (such as our Project ECHO case-based virtual learning series, conferences, webinars, and newsletters) improve the health of the patients within the participating Cardi-OH practices.

## Conclusions

CVD is one of the leading causes of death in the United States and many other countries and has key modifiable risk factors such as blood pressure control and smoking cessation. Primary care clinics continue to be a mainstay for people to work with clinicians to improve these modifiable risk factors and reduce cardiovascular morbidity and mortality. In addition, payers have influence over policies which can have great impacts on cardiovascular health. Our statewide collaborative model Cardi-OH which uses academic medical schools as the backbone organizations in partnership with payers and primary care clinics has the strong potential to improve cardiovascular health through the dissemination of evidence-based best practices into care, especially when tied with cardiovascular-focused QIPs which can measure and celebrate improvement in health outcomes. Using a collective impact model to develop a statewide cardiovascular health collaborative has a strong potential for other states to replicate statewide collaboratives for national improvements in cardiovascular health outcomes.
